# Supplementary material for: AKT isoforms have distinct hippocampal expression and roles in synaptic plasticity
Source: eLife. 2017 Nov 27;6:e30640. doi: 10.7554/eLife.30640 (PMC5722612; doi:10.7554/eLife.30640)
Supplement: Transparent reporting form [file elife-30640-transrepform.docx]

***eLife’s* transparent reporting form**

We encourage authors to provide detailed information *within their submission* to facilitate the interpretation and replication of experiments. Authors can upload supporting documentation to indicate the use of appropriate reporting guidelines for health-related research (see [EQUATOR Network](http://www.equator-network.org/%20)), life science research (see the [BioSharing Information Resource](https://biosharing.org/)), or the [ARRIVE guidelines](http://www.plosbiology.org/article/info:doi/10.1371/journal.pbio.1000412) for reporting work involving animal research. Where applicable, authors should refer to any relevant reporting standards documents in this form.

If you have any questions, please consult our Journal Policies and/or contact us: [editorial@elifesciences.org](mailto:editorial@elifesciences.org).

**Sample-size estimation**

- You should state whether an appropriate sample size was computed when the study was being designed
- You should state the statistical method of sample size computation and any required assumptions
- If no explicit power analysis was used, you should describe how you decided what sample (replicate) size (number) to use

Please outline where this information can be found within the submission (e.g., sections or figure legends), or explain why this information doesn’t apply to your submission:

We estimate our sample size based on previous experiment within our lab. Post-hoc power analysis has been used to determine sample size for experiments before they were conducted. However, because not all experiments are identical those conducted previously. In these cases, we designed the experiments based on those that most closely matched the design of the current experiment. Below are examples of how power from sample size were determined.

**-Immunostaining:** This is section is descriptive. We replicated the staining on three different WT animals. We have used this number because it has been considered conventional for experiments of this nature. To test if antibody was specific, we used two independent KO animals that were confirmed to be KO by PCR genotyping.

**-Biochemical western analysis (including SunSET):** For other biochemical analysis, we used 3-4 mice. For SunSET a minimum of 2 samples were prepared per mouse, then 4 mice/group.

Power (from Hoeffer et al. 2011)

Groups are: Slices treated with anisomycin or slices treated with vehicle.

Means of Puromycin/GAPDH ECL blot signal. N1,N2=4,4 (one mouse/sample/2 slices averaged); Means1,2=15.6,100; Effect Size: 0.6695; Sigma:2.231; FCrit:4.600; Power=0.88251

**-Western analyses (isolated hippocampal slices from mice 12 weeks of age)** Power analysis (from Wong et al. 2013):

Groups are: RCAN1 WT and RCAN1 KO. N1,N2=5,5 (one mouse/sample); Means of pCREB/tot CREB ECL blot signal. Means1,2=100,153.4; Effect Size: 1.1129; Sigma:23.99; FCrit:5.31176; Power=0.9768

**-Electrophysiology**: WT and KO mice are tested at the same day, and compounds and vehicles are used on hippocampal slices derived from same WT mice. We estimate the number of mice needed based on from Hoeffer et al. 2008 and we try to use ~2-3 slices/animal):

**For example:**

**HFS L-LTP in hippocampal brain slices:** Groups are: *Fkbp12cKO* and WT. N1,N2=10,10 (slices 2-3 each); Means of L-LTP (final 20 min), Means1,2=105.45,135.93; Effect Size: 1.4993; Sigma:20.33; FCrit:2.1009; Power=0.8866. The power in this case is really high, we have sampled our data sets and have found that N=5-6 to sufficient to generate sufficient power to perform the experiments.

In the figure legends, we describe the statistical test used, the number of slices used per experiment and how many mice were used.

**Replicates**

- You should report how often each experiment was performed
- You should include a definition of biological versus technical replication
- The data obtained should be provided and sufficient information should be provided to indicate the number of independent biological and/or technical replicates
- If you encountered any outliers, you should describe how these were handled
- Criteria for exclusion/inclusion of data should be clearly stated
- High-throughput sequence data should be uploaded before submission, with a private link for reviewers provided (these are available from both GEO and ArrayExpress)

Please outline where this information can be found within the submission (e.g., sections or figure legends), or explain why this information doesn’t apply to your submission:

-Electrophysiology: Only a max of two mice are tested/day (one WT and one KO), due to equipment limitations. But we try to get 2-3 slices per animal. Therefore, these experiments are replicated for 4-5 times (dependent # of mice/group).

- SunSET labeling: Only a max of two mice could be tested/day (one WT and one KO) due to equipment limitations. Therefore, these experiments are replicated 4-6 times.

Exclusion criteria: technical failures, for example should a power source fail or water droplet precipitate on slice. For experimental purposes, slices are excluded: for L-LTP, if the slices fail to show an 150% increase in long term potentiation based on Vmax for input/output curve; for LTD, if slices fail to show 15% depression from baseline.

**Statistical reporting**

- Statistical analysis methods should be described and justified
- Raw data should be presented in figures whenever informative to do so (typically when N per group is less than 10)
- For each experiment, you should identify the statistical tests used, exact values of N, definitions of center, methods of multiple test correction, and dispersion and precision measures (e.g., mean, median, SD, SEM, confidence intervals; and, for the major substantive results, a measure of effect size (e.g., Pearson's r, Cohen's d)
- Report exact p-values wherever possible alongside the summary statistics and 95% confidence intervals. These should be reported for all key questions and not only when the p-value is less than 0.05.

Please outline where this information can be found within the submission (e.g., sections or figure legends), or explain why this information doesn’t apply to your submission:

A general description of statistical approaches used is described in ‘Methods’.

Electrophysiology: Statistical analysis were performed using Repeated Measures ANOVA for timepoints post stimulation.

For biochemical analyses, we used ANOVA followed by post-hoc Tukey’s test in cases where more than two groups were compared (compound testing). In case where only two groups were tested, student’s T-test unpaired, two tailed was used when two groups were tested.

Where appropriate data were checked for normal distribution. Gibb’s outlier analysis were applied to all data sets.

(For large datasets, or papers with a very large number of statistical tests, you may upload a single table file with tests, Ns, etc., with reference to sections in the manuscript.)

**Group allocation**

- Indicate how samples were allocated into experimental groups (in the case of clinical studies, please specify allocation to treatment method); if randomization was used, please also state if restricted randomization was applied
- Indicate if masking was used during group allocation, data collection and/or data analysis

Please outline where this information can be found within the submission (e.g., sections or figure legends), or explain why this information doesn’t apply to your submission:

N/A

**Additional data files (“source data”)**

- We encourage you to upload relevant additional data files, such as numerical data that are represented as a graph in a figure, or as a summary table
- Where provided, these should be in the most useful format, and they can be uploaded as “Source data” files linked to a main figure or table
- Include model definition files including the full list of parameters used
- Include code used for data analysis (e.g., R, MatLab)
- Avoid stating that data files are “available upon request”

Please indicate the figures or tables for which source data files have been provided:
